# Knee arthrodesis: procedures and perspectives in the US from 1993 to 2011

**DOI:** 10.1186/s40064-016-3285-z

**Published:** 2016-09-20

**Authors:** Eric M. Lucas, Nicholas C. Marais, John D. DesJardins

**Affiliations:** Department of Bioengineering, Clemson University, 301 Rhodes Engineering Research Center, Clemson, SC 29634 United States

## Abstract

**Background:**

The incidence and prevalence of knee arthrodesis (fusion) in the United States is largely unknown, in spite of numerous case reports and review articles that have called attention to this life altering procedure.

**Purpose:**

This study was conducted to determine long-term knee arthrodesis incidence and patient populations, and to characterize the associated healthcare burden.

**Methods:**

The Nationwide Inpatient Sample was used to evaluate knee arthrodesis procedures performed in the United States between 1993 and 2011. Patient age, sex, and reimbursement method were evaluated along with hospital attributes. Procedural rates for individual demographics were calculated using population data from the US Census. Commonly occurring diagnoses and procedures in knee arthrodesis were compiled.

**Results:**

The annual number of reported knee arthrodesis procedures remained relatively unchanged between 1993 and 2011 (Mean 1014, Standard Deviation 113), but there was a small but significant decrease in the procedure rate when taking population changes into account. Over 80 % of patients were aged 45 or above. Approximately 65 % of patients utilized governmental payers for reimbursement. Nearly all of the procedures were performed in metropolitan area hospitals (92.5 %), and a significant majority performed in teaching hospitals (62 %).

**Conclusions:**

The low incidence of knee arthrodesis procedures reflects both clinician and patient antipathy for this undesirable surgery. Case studies continue to reflect an interest to improve methodology, but also suggest a significant number of patients that go untreated given the current state of the art. Future work should seek to quantify the prevalence of patients with a severely dysfunctional knee who might otherwise undergo arthrodesis, but opt against it given the significant quality of life issues associated with the procedure.

## Background

Historically, knee arthrodesis has had a wide range of clinical indications, including advanced osteoarthritis, posttraumatic osteoarthritis, rheumatoid arthritis, tuberculosis, poliomyelitis, and syphilis (Conway et al. 2004; Van Rensch et al. 2013). With the development and success of total knee replacement (TKR) surgery, along with advances in medicine eliminating the later stages of some diseases, the indications for arthrodesis have narrowed (Conway et al. 2004; Van Rensch et al. 2013; Brand 2010). Current indications for this treatment include bone or tissue damage, weakness or loss of the knee extensor mechanism, inadequate ligamentous constraint, substantial bone loss or defects, osteosarcoma, posttraumatic arthritis, arthrofibrosis, infection (Conway et al. 2004; MacDonald et al. 2006), and the failure of total knee replacements (Van Rensch et al. 2013; Jones et al. 2012; Rao et al. 2009; Bargiotas et al. 2006). However, the functional outcomes and quality of life after knee arthrodesis are generally low (Carr et al. 2016), with some studies showing that knee arthrodesis offers no significant protection against persistent infection or any substantial loss in pain (Röhner et al. 2015). Although this procedure has a long history of indication, little biomedical innovation in this area has enabled the wider acceptance of this procedure. This has made knee arthrodesis an undesirable, last resort option, used only in cases where other treatment options are inadequate or of unacceptable risk.

Ironically, clinical interest in knee arthrodesis procedures and outcomes remains constant, and there are a large number of published case reports and review articles (Somayaji et al. 2008) on the subject. Some of these reports have focused on the conflicting methods of achieving fusion, including external fixation (Ravi and Chaikof 2010; Spina et al. 2010; Roy et al. 2016) and internal fixation by means of intramedullary nails (Leroux et al. 2013; Lee et al. 2012; Razii et al. 2016) or dynamic compression plates (Roy et al. 2016; Lv et al. 2012). Clinical evidence indicates that internal fixation tends to be a simpler procedure; however, external fixation can be indicated in cases of substantial bone or tissue loss (Lee et al. 2012). While challenging conditions often undermine the success of knee arthrodesis procedures, advanced surgical techniques have been discribed to counteract some of the counterindications (Wood and Conway 2015). New methods of achieving fusion have been proposed (Voss 2001), including implantable prosthetics dedicated to knee arthrodesis (Rao et al. 2009; Angelini et al. 2013; Putman et al. 2013; Bartlett et al. 2011). These implants can serve as a rigid spacer in the absence of sufficient bone stock and can improve the surgical success rate, but they offer patients no functional advantages over traditional knee arthrodesis techniques.

The outcomes of knee arthrodesis are mixed. By salvaging the limb, knee arthrodesis can enable patients to ambulate without the use of assistive devices. This outcome facilitates mobility, and allows patients to maintain independence. However, the disadvantages of knee arthrodesis are notable—walking with an arthrodesis is more physically demanding than normal gait, and an immobilized knee can make activities of daily living such as sitting and tying ones shoes difficult. These chronic disadvantages have pressed some patients to elect for desarthrodesis, or reversal of a previous fusion, in spite of its high complication rate (Kuchinad et al. 2014; Ruggieri et al. 2010; Cho et al. 2008; Clemens et al. 2005; Kernkamp et al. 2016; Jauregui et al. 2016). In cases where radical revision surgery to save knee function is inadvisable or unethical, patients may even prefer to have the affected limb amputated rather than fused (Capozzi et al. 2009).

In spite of the frequently expressed clinical interest in knee arthrodesis, the only study that has estimated the incidence of the procedure, characterized the affected patients, or characterized the institutions where the procedure is performed was conducted using a Danish registry (Gottfriedson et al. 2016). One implication of this is that the true demand for development of superior treatments or techniques in the United States is unknown. Other orthopedic subpopulations in the United States have been well characterized using resources such as the National Hospital Discharge Survey and Nationwide Inpatient Sample, including patients undergoing knee, hip, and shoulder arthroplasty (Kim et al. 2011; Kurtz et al. 2005). That work has been influential, and has enabled further studies, such as estimations of economic burden (Kurtz et al. 2007), to build upon it.

We hypothesize that in spite of continued clinical interest in this procedure as a viable, all-be-it undesirable, end-stage option for patients, the number of procedures is not increasing over time. The purpose of this study was to estimate the historical incidence of knee arthrodesis in the United States, to characterize the affected patients and associated hospitals, and to give some perspectives on the clinical burden, demand, and potential opportunities for innovation in this clinical area.

## Methods

Data for this study was obtained from the Nationwide Inpatient Sample (NIS) (HCUP Nationwide Inpatient Sample (NIS), Healthcare Cost and Utilization Project (HCUP) 1997–2011), a database of inpatient discharge records produced by the Healthcare Cost and Utilization Project (HCUP), part of the Agency for Healthcare Research and Quality (AHRQ). The NIS is “the largest publicly available all-payer inpatient care database in the United States,” containing data from “approximately 8 million hospital stays each year.” Over one-hundred data elements are included with each discharge record, including patient age, patient sex, payment method, diagnoses and procedures performed, as well as characteristics of the admitting hospital. The NIS has a stratified, single stage cluster sample design, with individual hospitals as clusters and US region, urban or rural location, teaching status, ownership, and bed size as strata.

Annual data from the NIS is available as far back as 1988, but data for this study was limited to 1993–2011 due to the increased size and improved representativeness of the sample these years. The latest available dataset, from the year 2011, contains “all discharge data from 1045 hospitals located in 46 states, approximating a 20 % stratified sample of US community hospitals.” With an understanding of the sample design, weighting factors can be applied to sample data to produce national estimates. HCUP provides supplementary data for trends analyses spanning further back than 2002 to account for changes made to the sample design, and these adjustments were used for our analyses.

The NIS database was searched for patients undergoing knee arthrodesis as a primary or secondary procedure from 1993 through 2011 using *International Classification of Disease* (ICD-9-CM) procedural code 81.22, “Arthrodesis of the Knee.” We did not distinguish between external fixation, internal fixation, or other methods of fusion, as all methods of knee arthrodesis are included under the same code. This code was assumed to capture all incidences of knee arthrodesis within the sample, as it was well established for all years of our inquiry. In addition, this procedure was non-experimental and thus not prone to underreporting, and there are no similar procedural codes that were likely to be used in its place.

Demographic data including patient age, sex, and payment method were collected for all cases of knee arthrodesis. Patient age was grouped into six bins: ages 0–44, 45–54, 55–64, 65–74, 75–84, and 85 and older. Data collected on each discharge included the total number of procedures, length of stay, total charges, and the associated diagnoses and procedures performed. Each admitting hospital’s teaching status, location, and region of the country was also collected.

### Statistical methods

Nationwide estimates were produced for each year using the appropriate weighting factors for each observation, and standard errors are reported with consideration of the stratified sample design. In cases where knee arthrodesis was recorded as a secondary procedure, a frequency analysis was performed to determine the most commonly recorded primary procedures. Values of p < 0.05 were considered statistically significant in all cases.

A Poisson distribution, similar to the methods of Kurtz et al. (2005)., was assumed for the measures of total number of arthrodesis procedures, population based procedure rates, total number of procedures on each discharge record, and total length of stay. Procedural rates for each patient demographic (age, sex) were estimated using population figures provided by the United States Census Bureau. Intercensal estimates from July of each off-census year were used. Poisson regression analysis allowed for the use of age and sex as covariates in determining the rate and rate ratio of knee arthrodesis, and enabled us to calculate the procedure rate for each demographic group. There were no zero-values for total length of stay or total number of procedures on any discharge record due to study design; The Nationwide Inpatient Sample contains only inpatient discharge records, resulting in a 1 day minimum length of stay by definition, and we only assessed records containing a knee arthrodesis procedure, resulting in a minimum of one procedure on each discharge record. Zero-truncated Poisson regression was used to analyze the total number of procedures and the total length of stay on each discharge record to account for this. An analysis of the rate ratios across each year of study was used to determine the presence of significant annual trends.

Payment method, total hospital charges, hospital teaching status, hospital location, and hospital region were also investigated, and linear regression was used to determine trends across the years of study.

The diagnoses and other surgical procedures most commonly occurring in the presence of knee arthrodesis were determined using frequency analysis.

## Results

**Fig. 1:**
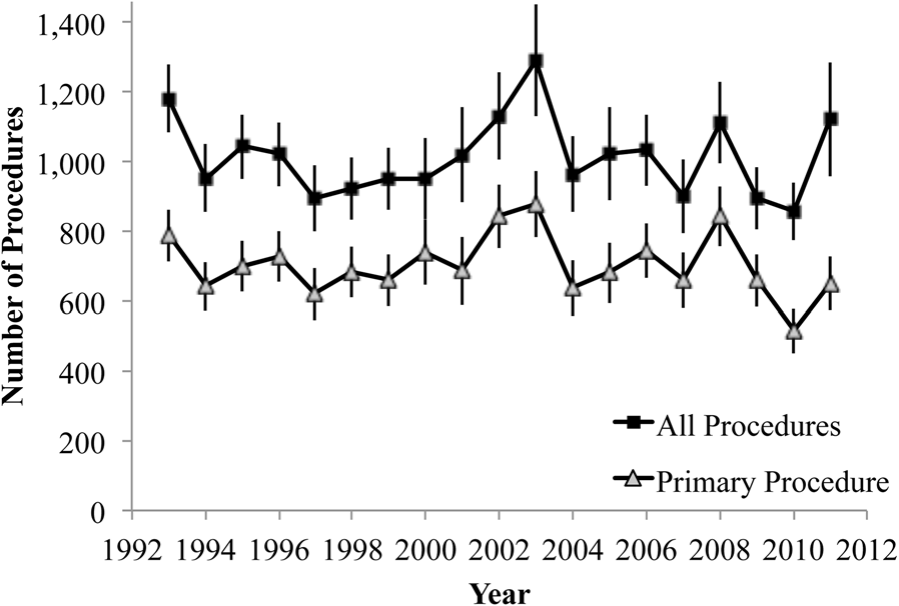
Estimates of the total number of knee arthrodesis procedures performed and the number coded as a Primary Procedure in the United States. Standard errors from NIS estimates are displayed

**Fig. 2:**
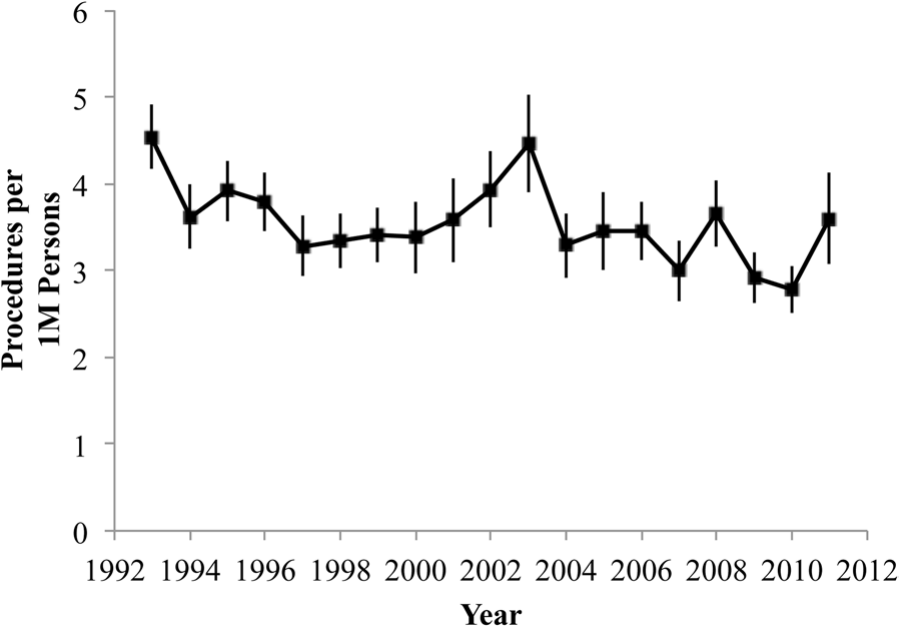
The rate of knee arthrodesis procedures performed per 1,000,000 people in the United States. Standard errors from NIS estimates are displayed

There were an average of 1014 knee arthrodesis procedures performed in the United States each year from 1993 and 2011 (Table 1), and there was no significant change in the absolute number of procedures performed over this time. When accounting for population growth, however, this resulted in a decrease in the per capita procedure rate observed during the years of this study (p < 0.01) (Fig. 2). Knee arthrodesis was recorded as the primary procedure in approximately 70 % of discharge records (Fig. 1).

**Table 1:** Estimated number of knee arthrodesis procedures in the United States from 1993 to 2011

	All procedures		Primary procedure	
	Estimate	SE	Estimate	SE
1993	1180	97	789	75
1994	952	98	642	72
1995	1044	93	700	72
1996	1021	91	729	74
1997	895	95	621	75
1998	922	88	684	73
1999	952	90	659	73
2000	1021	117	740	93
2001	928	139	687	98
2002	1131	124	845	92
2003	1293	162	879	97
2004	963	109	639	81
2005	1022	133	682	87
2006	1032	101	745	80
2007	902	107	660	79
2008	1111	115	843	85
2009	895	89	661	76
2010	858	83	516	65
2011	1122	163	652	77
Avg.	1013	110	704	80

**Fig. 3:**
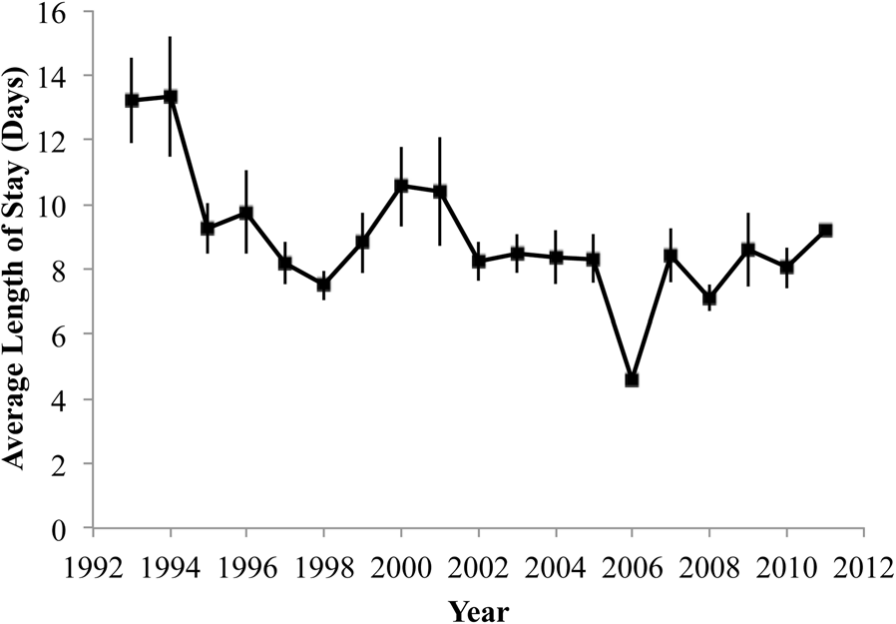
Average estimated length of stay for patients undergoing knee arthrodesis. Standard errors from NIS estimates are displayed

Both age and sex had a significant impact on the per capita rate of knee arthrodesis procedures (Table 2). The procedure rate was significantly higher for men of each age group, and the rate significantly increased with age for both sexes until at least age 84 (Table 3). Both the total number and per capita rate of knee arthrodesis procedures for patients aged 0–44 dropped significantly over the years of study (p < 0.001 and p < 0.001). The total number of procedures in patients aged 45–54 and 55–64 rose (p < 0.01), but the per capita rate remained constant. There was no change in the total number of procedures in patients aged 65–74 and 75–84, but the per capita rate dropped (p = 0.02). The average length of hospital stay was significantly influenced by age (p < 0.001), but not sex, increasing by an average of 1.04 days (95 % CI 1.03–1.05) with each increase in age group. Overall, there was a significant decrease in the average length of hospital stay between 1993 and 2011 (p < 0.01) (Fig. 3).

**Table 2:** Results of Poisson regression analysis—rate ratios by age, gender

	Knee arthrodesis rate ratios	
	Rate ratio	95 % CI
Gender		
Male	1.00	–
Female	0.83	(0.80, 0.85)
Age group		
0–44	1.00	–
45–54	3.97	(3.78, 4.17)
55–64	7.32	(6.99, 7.66)
65–74	11.64	(11.13, 12.16)
75–84	15.00	(14.32, 15.71)
85+	*	*

* Knee arthrodesis was performed in some patients above the age of 84, but the frequency of occurrence in the NIS was not sufficient to allow for analysis

**Table 3:** Rate of knee arthrodesis procedures per 1,000,000 persons

Rate of knee arthrodesis per 1,000,000 persons		
Age group	Male (95 % Cl)	Female (95 % CI)
0–44	1.13 (1.09, 1.17)	0.93 (0.90,0.97)
45–54	4.42 (4.25, 4.59)	3.66 (3.52, 3.80)
55–64	8.10 (7.82, 8.38)	6.70 (6.47, 6.94)
65–74	13.06 (12.64, 13.50)	10.81 (10.46, 11.17)
75–84	16.82 (16.22, 17.44)	13.92 (13.44, 14.42)
85+	*	*

* Knee arthrodesis was performed in some patients above the age of 84, but the frequency of occurrence in the NIS was not sufficient to allow for analysis

**Fig. 4:**
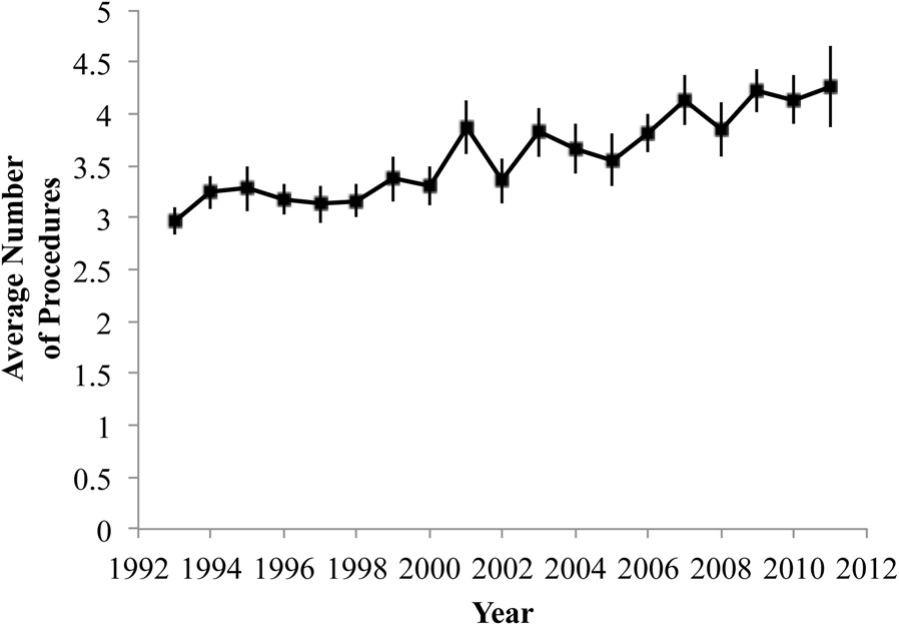
The average number of procedures recorded on each discharge record containing a knee arthrodesis procedure. Standard errors from NIS estimates are displayed

The average number of procedures performed alongside knee arthrodesis was significantly influenced by age (p = 0.014), increasing by an average of 1.03 (95 % CI 1.01–1.05) procedures per age group. The average number of procedures on each discharge record rose over the years analyzed at a rate of 0.067 procedures per year (p < 0.01) (Fig. 4). The diagnoses most commonly appearing on discharge records with a knee arthrodesis are unspecified hypertension, infection and inflammatory reaction due to internal joint prosthetic, and acute posthemorrhagic anemia (ICD-9-CM 4019, ICD-9-CM 99666, and ICD-9-CM 2851, respectively). The procedures most commonly appearing alongside knee arthrodesis are packed cell transfusion and arthrotomy for removal of a prosthesis without replacement (ICD-9-CM 9904 and ICD-9-CM 8006, respectively).

**Fig. 5:**
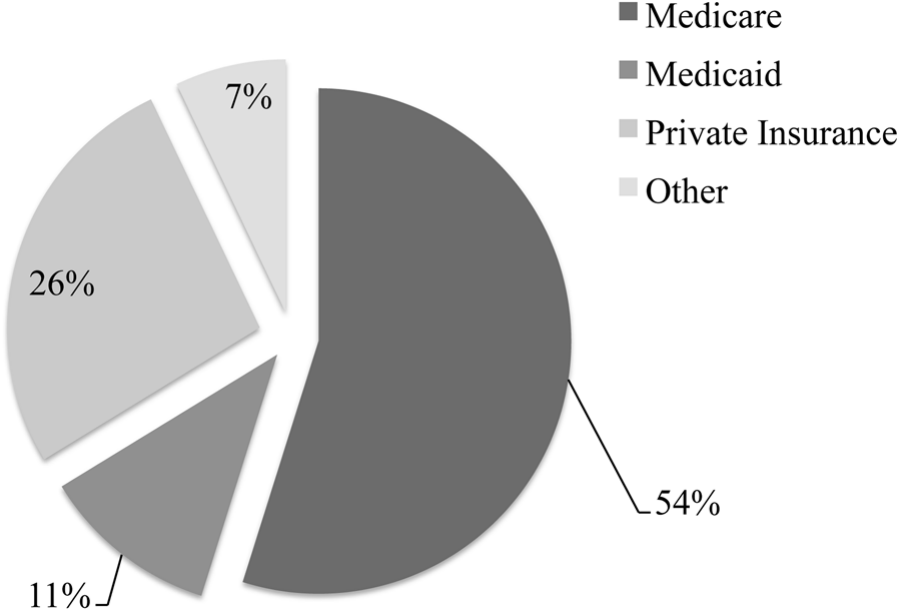
Breakdown of reimbursement method, by payer, for discharges containing a knee arthrodesis procedure

**Fig. 6:**
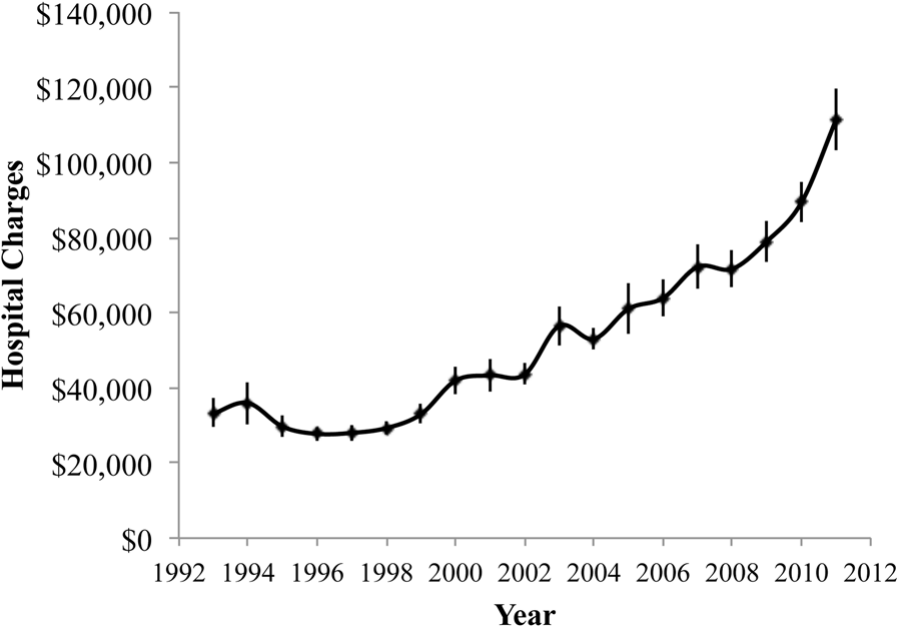
Average total hospital charges for patients undergoing knee arthrodesis

Medicare served as the payer for a majority (54 %; 10,361 of 19,269) of knee arthrodesis procedures, while private insurance was the next most frequently used payment method (26 %; 5056 of 19,269) (Fig. 5). No significant changes in these rates were observed in the range of years investigated. The average hospital charge for patients undergoing knee arthrodesis rose significantly (p < 0.001) over the years of study, more than tripling from a mean of $33,358 in 1993 to a mean of $111,312 in 2011 (Fig. 6).

Nearly all knee arthrodesis procedures were performed in metropolitan areas (92 %; 17,676 of 19,269), and a large majority (62 %; 11,859 of 19,269) were performed in teaching hospitals.

**Fig. 7:**
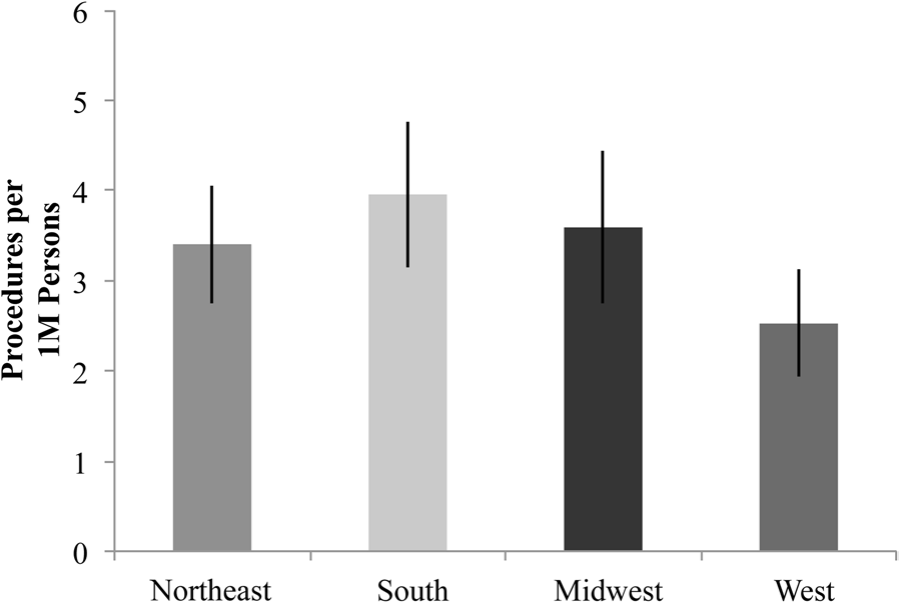
Distribution of knee arthrodesis procedure rate, by region of the country

The location of knee arthrodesis procedures was also analyzed by region of the United States. We followed the regional divisions of the NIS: Northeast, Midwest, South, and West. When accounting for population differences, the procedure rate was significantly smaller in the West than in any other region (p = 0.05) (Fig. 7).

## Discussion

The success of primary and revision total knee arthroplasty has significantly reduced the indication for knee arthrodesis as a primary treatment of dysfunctional knee pathology (Conway et al. 2004), but has not completely eliminated it. Arthrodesis is indicated in patients with deficient or missing extensor mechanisms, as total knee replacement designs cannot compensate for the loss of function and stability and the risk of graft transfer may be unacceptably high (Capozzi et al. 2009). However, knee arthrodesis often follows failed knee arthroplasty, as determined by our procedure analysis, and it could be hypothesized that the incidence of arthrodesis will increase as the number of revision arthroplasty procedures increases. However, this must be balanced against patients increasing expectations for higher quality of life, function, and mobility, for which arthrodesis technology has not kept pace. These trends, when combined, predict that there is a growing number of patients whose needs will be unmet through current arthrodesis methods.

Limitations of this study can first be extended to the comprehensive nature of the Nationwide Inpatient Sample. While this database is the largest inpatient database available, it may not be perfectly representative of the intended population. Also, this study was unable to provide further depth, as specific patient procedures cannot be tracked and the extent of injury that requires arthrodesis is not listed.

While knee fusion is rarely a preferred outcome, this study found that the number of arthrodesis procedures performed in the United States has not significantly changed over the past two decades. A small reduction in the total number of procedures may have been prevented by population growth, as the per capita rate of knee arthrodesis procedures has fallen or remained unchanged for each individual age/gender demographic group. It is noted that arthrodesis rates do not mirror increases in total knee revision rates. Given the steady number of knee arthrodesis procedures, it is likely that reconstructive knee replacement is already performed in as many cases as is feasible. It is also possible that high risk reconstructions are performed too often, and that knee arthrodesis should be attempted earlier in some cases to preserve bone stock and ensure the greatest chance of success (Knutson et al. 1985). The continued use of knee arthrodesis as treatment, in spite of its undesirability, indicates an otherwise unmet need in the affected patients. This merits a closer look by clinicians, researchers, and medical device manufacturers.

We found that patients who do undergo arthrodesis are generally older, with over 80 % of all patients above the age of 45, and over 41 % of patients above the age of 65. Age alone does not fully explain the prominence of Medicare as a payment method, however. Assuming that all patients above the standard eligibility age of 65 utilized Medicare as a primary payment method, there was still a significant proportion (12.3 %) of younger patients utilizing it. These patients most likely received Social Security Disability benefits prior to their knee arthrodesis procedure, an indication of the debilitating nature of the knee dysfunction and injuries that are present in patients who undergo the procedure. The arthrodesis age demographic found in this study is reminiscent of historical TKR patient populations, when TKR systems and materials were only needed to outlive the patient, and patient demands on implant performance was not has high.

We also found that a significant majority of arthrodesis patients obtained treatment in metropolitan areas and at teaching hospitals. This may be indicative of the complicated nature of the procedure, the severity of the negative side effects, and the severity of the potential complications. It is possible that only a small number of orthopedic surgeons perform the majority of these procedures, but this was not explored in the current study. Patients appear to be much more likely to either receive referrals or actively seek out specialists in knee reconstruction.

Arthrodesis can enable a patient to ambulate independently, but permanent rigid knee extension has severe functional limitations and can make sitting uncomfortable or impossible in public spaces. Simple activities of daily living, such as bathing or tying one’s shoes, can become difficult or impossible. These patients often face poor alternatives, however, including transfemoral amputation and resection knee arthroplasty (Jones et al. 2012). Some patients may voice a preference for amputation, but functional outcomes are typically worse than those of arthrodesis (Conway et al. 2004). And while resection arthroplasty both salvages the limb and enables a patient to bend the knee and sit comfortably, patients are typically unable to walk. At least one knee implant type design has been developed to address this population (Bartlett et al. 2011), but it offers patients no functional advantage over traditional fusion methods. When compared against these options, arthrodesis often offers the best-of-the-worst combination of function and risk. A Through a Voice-Of-Customer survey with orthopedic surgeons experienced in knee reconstruction and arthrodesis, we estimate that only one quarter to one third the number of patients who are suitable candidates for knee arthrodesis actually undergo the procedure, often in an attempt to avoid its limitations.

This study shows that there is a relatively small, but significant number of knee arthrodesis procedures performed each year, and that this number has remained relatively steady over the past two decades. The average cost of these discharges has more than tripled (Fig. 6) in that time, even as the average length of stay has gone down (Fig. 3). The rising hospital costs for knee arthrodesis outpace the general trend of rising costs for all procedures, as well as for Primary and Revision TKR. The high level of interest in knee arthrodesis, as evidenced by the number of published case study articles, is likely due to both the undesirability of the procedure and the significant number of affected or candidate patients seen by publishing/researching clinicians.

Looking forward, we expect the number of knee arthrodesis procedures to remain steady and the associated costs to continue to rise. These trends could be affected by the development of technological or procedural advances to address the affected patient subpopulation. This could include implants specifically designed to provide limited knee function or modular implant systems to improve outcomes following massive knee reconstruction. The biomechanical study of knee arthrodesis patient functional demands and the characterization of hospitals where it is performed provides researchers, clinicians, and medical device manufacturers with information critical to assessing the demand for new treatment approaches and implant design in this area.

## References

[CR1] Angelini A, Henderson E, Trovarelli G, Ruggieri P (2013). Is there a role for knee arthrodesis with modular endoprostheses for tumor and revision of failed endoprostheses?. Clin Orthop Relat Res.

[CR2] Bargiotas K, Wohlrab D, Sewecke JJ, Lavinge G, DeMeo PJ, Sotereanos NG (2006). Arthrodesis of the knee with a long intramedullary nail following the failure of a total knee arthroplasty as the result of infection. J Bone Joint Surg Am.

[CR3] Bartlett W, Vijayan S, Pollock R (2011). The Stanmore knee arthrodesis prosthesis. J Arthroplasty.

[CR4] Brand RA (2010). Arthrodesis of the knee joint. F. H. Moore and I. S. Smillie. CORR;13:215–221. Clin Orthop Relat Res.

[CR5] Capozzi JD, Rhodes R, Chen D (2009). Discussing treatment options. J Bone Joint Surg Am.

[CR6] Carr JB, Werner BC, Browne JA (2016). Trends and outcomes in the treatment of failed septic total knee arthroplasty: comparing arthrodesis and above-knee amputation. J Arthroplasty.

[CR7] Cho SH, Jeong ST, Park HB, Hwang SC, Kim DH (2008). Two-stage conversion of fused knee to total knee arthroplasty. J Arthroplasty.

[CR8] Clemens D, Lereim P, Holm I, Reikerås O (2005). Conversion of knee fusion to total arthroplasty: complications in 8 patients. Acta Orthop.

[CR9] Conway JD, Mont MA, Bezwada HP (2004). Arthrodesis of the knee. J Bone Joint Surg Am.

[CR10] Gottfriedson TB, Schroeder HM, Odgaard A (2016). Knee arthrodesis after failure of knee arthroplasty: a nationwide register-based study. J Bone Joint Surg Am.

[CR11] HCUP Nationwide Inpatient Sample (NIS), Healthcare Cost and Utilization Project (HCUP). 1997–2011. Agency for Healthcare Research and Quality, Rockville, MD. www.hcup-us.ahrq.gov/nisoverview.jsp

[CR12] Jauregui JJ, Buitrago CA, Pushilin SA, Browning BB, Mulchandani NB, Maheshwari AV (2016). Conversion of a surgicall arthrodesed knee to a total knee arthroplasty—is it worth it? A Meta-Analysis. J Arthroplasty.

[CR13] Jones RE, Russell RD, Huo MH (2012). Alternatives to revision total knee arthroplasty. J Bone Joint Surg Br.

[CR14] Kernkamp WA, Verra WC, Pijls BG, Schoones JW, van der Linden HM, Nelissen RG (2016). Conversion from knee arthrodesis to arthroplasty: systematic review. Int Othop.

[CR15] Kim SH, Wise BL, Zhang Y, Szabo RM (2011). Increasing incidence of shoulder arthroplasty in the United States. J Bone Joint Surg Am.

[CR16] Knutson K, Lindstrand A, Lidgren L (1985) Arthrodesis for failed knee arthroplasty. A report of 20 cases 10.1302/0301-620X.67B1.3968143 3968143

[CR17] Kuchinad R, Fourman MS, Fragomen AT, Rozbruch SR (2014). Knee arthrodesis as limb salvage for complex failures of total knee arthroplasty. J Arthroplasty.

[CR18] Kurtz SM, Mowat F, Ong K, Chan N, Lau E, Halpern M (2005). Prevalence of primary and revision total hip and knee arthroplasty in the United States from 1990 through 2002. J Bone Joint Surg Am.

[CR19] Kurtz SM, Ong KL, Schmier J (2007). Future clinical and economic impact of revision total hip and knee arthroplasty. J Bone Joint Surg Am.

[CR20] Lee S, Jang J, Seong SC, Lee MC (2012). Distraction arthrodesis with intramedullary nail and mixed bone grafting after failed infected total knee arthroplasty. Knee Surg Sports Traumatol Arthrosc.

[CR21] Leroux B, Aparicio G, Fontanin N (2013). Arthrodesis in septic knees using a long intramedullary nail: 17 consecutive cases. Orthop Traumatol Surg Res.

[CR22] Lv C, Tu C, Min L, Duan H (2012). Allograft arthrodesis of the knee for giant cell tumors. Orthopedics.

[CR23] MacDonald JH, Agarwal S, Lorei MP, Johanson NA, Freiberg AA (2006). Knee arthrodesis. J Am Acad Orthop Surg.

[CR24] Putman S, Kern G, Senneville E, Beltrand E, Migaud H (2013). Knee arthrodesis using a customised modular intramedullary nail in failed infected total knee arthroplasty. Orthop Traumatol Surg Res.

[CR25] Rao MC, Richards O, Meyer C, Jones RS (2009). Knee stabilisation following infected knee arthroplasty with bone loss and extensor mechanism impairment using a modular cemented nail. Knee.

[CR26] Ravi S, Chaikof EL (2010). Biomaterials for vascular tissue engineering. Regen Med.

[CR27] Razii N, Abbas AM, Kakar R, Agarwal S, Morgan-Jones R (2016). Knee arthrodesis with a long intramedullary nail as limb salvage for complex periprosthetic infections. Eur J Orthop Surg Tramatol.

[CR28] Röhner E, Windisch C, Nuetzmann K, Rau M, Arnhold M, Matziolis G (2015). Unsatisfactory outcome of arthrodesis performed after septic failure of revision total knee arthroplasty. J Bone Joint Surg Am.

[CR29] Roy AC, Albert S, Gouse M, Inia DB (2016). Functional outcome of knee arthrodesis with a monorail external fixator. Strateg Trauma Limb Reconstr.

[CR30] Ruggieri P, Kasimatis G, Errani C, Bosco G, Mercuri M (2010). Desarthrodesis and prosthetic reconstruction of the knee after resection of bone tumors. J Surg Oncol.

[CR31] Somayaji HS, Tsaggerides P, Ware HE, Dowd GSE (2008). Knee arthrodesis—a review. Knee.

[CR32] Spina M, Gualdrini G, Fosco M, Giunti A (2010). Knee arthrodesis with the Ilizarov external fixator as treatment for septic failure of knee arthroplasty. J Orthop Traumatol.

[CR33] Van Rensch PJH, Van de Pol GJ, Goosen JHM, Wymenga AB, De Man FHR (2013). Arthrodesis of the knee following failed arthroplasty. Knee Surg Sports Traumatol Arthrosc.

[CR34] Voss F (2001). A new technique of limb salvage after infected revision total knee arthroplasty—artificial fusion. J Arthroplasty.

[CR35] Wood JH, Conway JD (2015). Advanced concepts in knee arthrodesis. World J Orthop.

